# Angiomyxolipoma of the hard palate: a rare case report

**DOI:** 10.11604/pamj.2026.53.2.50579

**Published:** 2026-01-05

**Authors:** Samir Mainassara Chekaraou, Kadre Alio Kadre Ousmane, Abdoul Hafizou Rabe Amani, Abdoul Majid Habibou, Mahamadou Dandy

**Affiliations:** 1 Odontology Department, Niamey Military Hospital, Niamey, Niger,; 2 Oral and Maxillofacial Service, General Reference Hospital, Niamey, Niger

**Keywords:** Lipoma, angiomyxolipoma, surgical excision, histology, case report

## Abstract

Lipoma is a common, benign soft tissue neoplasm consisting of mature adipocytes. Other variants exist, notably angiomyxolipoma, also referred to as vascular myxolipoma, which is an exceedingly rare benign lipomatous tumor characterized by the intimate admixture of three components: mature adipose tissue, paucicellular myxoid stroma, and a prominent vascular network. We present the case of a 55-year-old female patient who presented with a palatal swelling. Histopathological examination after surgical excision revealed an angiomyxolipoma. No abnormalities were noted after one year. Angiomyxolipoma of the oral cavity is an exceptionally rare benign neoplasm, with only a handful of well-documented cases involving the buccal mucosa, floor of mouth, tongue, and lip. Despite its rarity, recognition is important because the lesion can closely mimic other lipomatous or myxoid tumors, including malignant entities such as myxoid liposarcoma.

## Introduction

Lipoma is a common, benign soft tissue neoplasm consisting of mature adipocytes usually found in the subcutaneous tissue of the trunk, proximal limbs, thigh, and neck. Twenty percent (20%) of lipomas are found in the head and neck region, with 1-5% found in the oral cavity [1]. Other variants exist, notably angiomyxolipoma. Angiomyxolipoma (AML), also referred to as vascular myxolipoma, is an exceedingly rare benign lipomatous tumor characterized by the intimate admixture of three components: mature adipose tissue, paucicellular myxoid stroma, and a prominent vascular network (thin- and thick-walled vessels). This entity was first recognized in the subcutaneous soft tissues in the early 1990s and described for the first time in the oral cavity in 2011 [2,3]. Our case is the first described at the level of the hard palate.

## Patient and observation

**Patient information:** a 55-year-old female patient in good general health consulted for a painless palatal swelling that had been developing for 5 years at the odontology department of Niamey Military Hospital.

**Figure 1:**
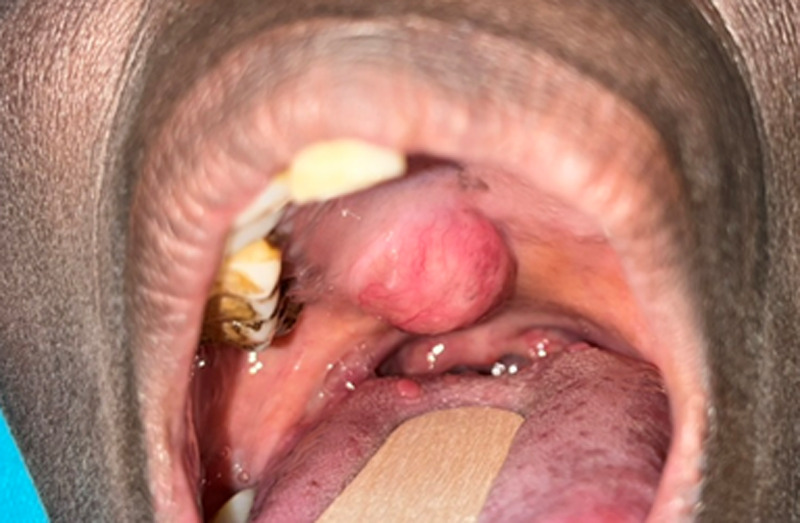
intraoral view of the lesion

**Clinical finding:** the extraoral examination was unremarkable on inspection and palpation, with no cervical lymphadenopathy. Intraoral examination revealed a 5 cm x 2 cm yellowish-red swelling on the hard palate that was firm and depressible (Figure 1). Panoramic radiography was unremarkable.

**Figure 2:**
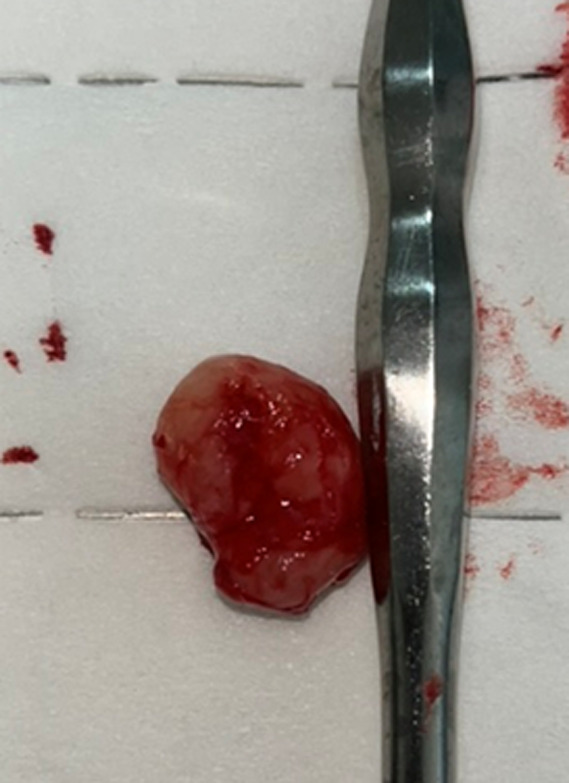
view of the lesion after surgical excision

**Therapeutic intervention:** under local anesthesia, careful dissection allowed surgical excision of the lesion, which was sent for pathological examination (Figure 2). The patient was seen again after one week with an uncomplicated postoperative recovery.

**Differential diagnosis:** at this stage, several diagnostic hypotheses were considered, including lipoma, leiomyoma, and pleomorphic adenoma.

**Diagnostic assessment:** pathological examination revealed a composite tissue proliferation consisting of more or less rounded adipose cells, capillaries with myxoid components, and fibromas, confirming the diagnosis of angiomyxolipoma.

**Follow-up:** the patient was seen one year later with no recurrence.

**Patient's perspective:** the patient was satisfied with the immediate postoperative period.

**Informed consent:** written informed consent for publication of the medical report and for all the interventions, was obtained from the patient.

## Discussion

Angiomyxolipoma (vascular myxolipoma) is a rare form of lipoma with only a dozen cases that have been documented across all anatomical sites, characterized by proliferation of adipose tissue with myxoid stroma and numerous dilated vascular channels [4]. Oral cavity involvement is exceptional. Only four cases have been described in the literature (tongue, upper lip, buccal mucosa) [5-8]. Our case is the first on the hard palate. Patient age ranges from children to elderly adults, with no clear ethnic predilection. Angiomyxolipoma in the mouth typically presents as a slow-growing, well-circumscribed, painless nodule. Depending on location, mass effect may cause functional disturbances: swallowing or speech difficulty, tongue displacement, or cosmetic/functional discomfort. Buccal mucosa lesions appear as submucosal swellings with preserved overlying mucosa [4,6,7].

Radiographically, AML may appear on CT and MRI as a well-defined mass with mixed fatty and myxoid/vascular components, particularly in the floor of the mouth. Imaging is helpful to assess the extent and relation to adjacent structures, but diagnosis remains histological [9,10]. Immunohistochemistry and histopathology are sufficient to ensure a correct diagnosis; the positivity of the spindle cells of the myxoid areas for CD34 and in the vascular endothelium for CD34 and CD31 are essential in correct differential diagnosis [9,10].

The differential diagnosis of angiomyxolipoma is made with myxolipoma, spindle cell/pleomorphic lipoma with myxoid change, angiolipoma, angiomyolipoma, and myxoid liposarcoma [5]. The treatment of choice is complete local excision with a margin by surgery, as in our case. CO_2_ laser excision with primary closure [5]. These lesions require monitoring at three, six months up to one year, even though no recurrence or malignant transformation has been noted in the literature [9,10].

## Conclusion

Angiomyxolipoma of the oral cavity is an exceptionally rare benign neoplasm, with only a handful of well-documented cases involving the buccal mucosa, floor of mouth, tongue, and lip. Despite its rarity, recognition is important because the lesion can closely mimic other lipomatous or myxoid tumors, including malignant entities such as myxoid liposarcoma. Histopathology, supported by immunohistochemistry, remains the cornerstone of diagnosis, with the typical triad of mature adipose tissue, paucicellular myxoid stroma, and abundant vasculature. Complete local excision is curative, with no recurrences or malignant transformation reported to date. Further accumulation of case reports and ideally multicenter analyses will be needed to better define its biological spectrum, refine diagnostic criteria, and ensure accurate distinction from its histological mimics.
